# Factors Associated with Hormonal and Intrauterine Contraceptive Use among HIV-Infected Men and Women in Lilongwe, Malawi: A Cross-Sectional Study

**DOI:** 10.1155/2016/5429316

**Published:** 2016-08-24

**Authors:** Jennifer H. Tang, Sam Phiri, Wingston Ng'ambi, Jamie W. Krashin, Linly Mlundira, Thom Chaweza, Bernadette Samala, Hannock Tweya, Mina C. Hosseinipour, Lisa B. Haddad

**Affiliations:** ^1^UNC Project-Malawi, Kamuzu Central Hospital, 100 Mzimba Road, Private Bag Box A-104, Lilongwe, Malawi; ^2^University of North Carolina School of Medicine, Department of Obstetrics & Gynecology, University of North Carolina at Chapel Hill, 101 Manning Drive, CB No. 7570, Chapel Hill, NC 27599-7570, USA; ^3^The Lighthouse Trust, Kamuzu Central Hospital, 100 Mzimba Road, Lilongwe, Malawi; ^4^University of North Carolina School of Medicine, Department of Medicine, Chapel Hill, NC 27599-3368, USA; ^5^The International Union against Tuberculosis and Lung Disease, 75006 Paris, France; ^6^Emory University School of Medicine, Department of Gynecology and Obstetrics, Emory University, 49 Jesse Hill Jr. Drive, Atlanta, GA 30303, USA

## Abstract

*Background*. Understanding the factors associated with the use of hormonal and intrauterine contraception among HIV-infected men and women may lead to interventions that can help reduce high unintended pregnancy rates.* Materials and Methods*. This study is a subanalysis of a cross-sectional survey of 289 women and 241 men who were sexually active and HIV-infected and were attending HIV care visits in Lilongwe, Malawi. We estimated adjusted prevalence ratios (PRs) to evaluate factors associated with hormonal and intrauterine contraceptive use for men and women in separate models.* Results and Discussion*. 39.8% of women and 33.2% of men (*p* = 0.117) reported that they were using hormonal or intrauterine contraception at last intercourse. Having greater than 3 children was the only factor associated with hormonal and intrauterine contraceptive use among men. Among women, younger age, not wanting a pregnancy in 2 years, being with their partner for more than 4 years, and being able to make family planning decisions by themselves were associated with hormonal and intrauterine contraceptive use.* Conclusions*. The men and women in our study population differed in the factors associated with hormonal and intrauterine contraceptive use. Understanding these differences may help decrease unmet FP needs among HIV-infected men and women.

## 1. Background

At the end of 2014, an estimated 36.9 million people were living with HIV. Seventy percent (25.8 million) of these HIV-infected people lived in Sub-Saharan Africa [1]. Malawi has one of the highest HIV prevalence rates in the world, with an estimated 1.1 million people living with HIV [2]. Its overall prevalence rate is 10.6%, although the prevalence rate is almost 5% higher among women (12.9%) than among men (8.1%) due to their increased vulnerability to HIV acquisition [3].

In July 2011, Malawi initiated a four-pronged strategy for prevention of maternal-to-child transmission of HIV. Prong 2 focuses on the reduction of unplanned or unintended pregnancies among HIV-infected women and included the integration of family planning (FP) provision into HIV care via HIV provider-initiated FP [4]. The strategy emphasized dual protection, stating that “condoms alone are not enough for family planning as they have to be used very consistently.” More recently, Malawi published its HIV Prevention Strategy for 2015–2020, which has set a target of reducing unplanned or unintended pregnancies among HIV-infected women from 42,645/year in 2015 to 39,565/year by 2020, a 7.2% reduction [5]. It aims to meet this goal by increasing the contraceptive prevalence rate (CPR) and eliminating unmet need for FP among all women, which are currently at 35% and 18.5%, respectively [3]. The unmet need for FP is likely to be even higher among HIV-infected women, as a recent survey of HIV-infected women found that 68% of their last pregnancies were unintended, compared with the national unintended pregnancy rate of 45% [3, 6].

To increase contraceptive use among HIV-infected couples, we must first understand the characteristics of HIV-infected persons who are and are not using contraceptives in their relationship. Multiple studies in Sub-Saharan Africa have assessed factors associated with modern contraceptive use among HIV-infected women, which include the following: younger age [7, 8], higher education [7, 9, 10], higher income or socioeconomic status [7, 10], being married or in a committed relationship [8, 10], living in an urban setting [9], having more children [7–12], both partners not wanting more children [13], current use of antiretroviral therapy (ART) [12, 14, 15], disclosure to partner about HIV-infected status [8, 12, 16], having a regular sexual partner [9, 10, 17], talking with the partner about FP [9, 10], and being healthier by assessment by either CD4+ count or hemoglobin levels [8].

However, all the previously cited studies except for Polis et al.'s study included condoms as a contraceptive in their analyses. As condoms require very consistent use to be effective, they are considered to be only “moderately effective” or Tier 3 in their contraceptive effectiveness, as compared to oral contraceptives (OC) and the depot medroxyprogesterone acetate (DMPA) injection, which are “effective” or Tier 2, and the subdermal implant and the copper intrauterine device (IUD), which are “very effective” or Tier 1 [18]. In addition, condom use may be primarily used to decrease acquisition or transmission of HIV or sexually transmitted infections rather than being used for contraceptive purposes. Finally, none of these studies surveyed HIV-infected men about their contraceptive use. Therefore, the objective of this study was to evaluate factors that are associated with hormonal or intrauterine contraceptive use (the most effective nonpermanent contraceptive methods) among both HIV-infected women and men.

## 2. Materials and Methods

This cross-sectional study was approved by Malawi National Health Sciences Research Committee, the Emory University Institutional Review Board, and the University of North Carolina Institutional Review Board. It enrolled participants over three months at two Lighthouse Trust clinics in Lilongwe, Malawi, from 26 September 2013 to 20 December 2013. The Lighthouse Trust is a registered public trust that works closely with the Malawi Ministry of Health to operate two large integrated HIV testing, treatment, and care clinics in Lilongwe. One clinic (Lighthouse) is based in the campus of Kamuzu Central Hospital and the other (Martin Preuss Clinic or MPC) is based at Bwaila Hospital under the Lilongwe District Health Office. Together, the two clinics care for over 23,000 patients on ART and over 2,000 patients who are not yet clinically eligible for ART. The Lighthouse began integrating FP services into its clinics in 2010, and, by the time of this study, it offered the following contraceptives on-site: condoms, OC, the DMPA injection, subdermal implants, and the copper IUD. MPC began its integration in February 2013 and offered condoms, OC, and DMPA on-site during the time of the study; for implant and IUD, it referred women to the Bwaila FP Clinic, which is located in building adjacent to MPC. Since male and female sterilization were not available at either clinic, referrals were made by both clinics for these services [19].

Study participants were recruited from the general waiting rooms of both clinics. Those who were interested in the study were invited into a private room, where they would be screened for eligibility into the study by a study research assistant. Potential participants were eligible if they (1) were between the ages of 18 and 45 years, (2) spoke Chichewa (the most commonly spoken local language) fluently, (3) had a sexual partner within the past 6 months, (4) had a documented HIV positive status, and (5) were a registered client at either Lighthouse or MPC. After confirming study eligibility, a research assistant would then undergo the informed consent process with the potential participant and obtain written consent if they agreed to enroll. Of note, the men and women in the study may have had partners who were also enrolled in the study, but these participants were not enrolled as couple dyads and completed their surveys independently of their partners.

Once enrolled, the study participant completed a face-to-face paper-based questionnaire administered by one of our two research assistants, with 160 questions for women and 130 questions for men. The questionnaire included information on the following: (1) demographics, (2) HIV and STI history, (3) condom use, (4) sexual history and current sexual behavior, (5) fertility preferences and pregnancy history, (6) contraceptive knowledge, attitudes, and use, (7) communication, and (8) ART knowledge and use. Questions were a compilation of original study questions and questions used in the Malawi 2010 Demographic and Health Survey [3]. Focus group discussions among men and women at the clinic conducted prior to this study also helped inform questionnaire development [manuscript under review].

A study database in Microsoft Access 2007 (Microsoft, Redmond, WA, USA) was created for the data entry and management. All data were double-entered and validated using predetermined queries. Stata 11.1 (StataCorp LP, College Station, TX, USA) was used for the statistical analysis. Based on the attendance numbers at the two clinics and our budget, we determined that we could enroll a convenience sample of up to 600 participants during our 3 months of recruitment. For this analysis, we excluded participants who had missing data on contraceptive use or who had used a permanent method of contraception (bilateral tubal ligation or vasectomy) at last intercourse.

Multivariable modified Poisson regression analysis with robust variance was used to estimate the unadjusted and adjusted prevalence ratios (PRs) and 95% confidence intervals (CIs) for all exposure variables selected for inclusion in our models [20, 21]. Our outcome variable was use of hormonal or intrauterine methods (OC, DMPA injection, implant, and the IUD) at last intercourse. Since we hypothesized that the factors that influence hormonal and intrauterine contraceptive use would differ by gender, we ran two separate models: one for female participants and one for male participants. To select our exposure variables for both models, we reviewed the literature and identified 18 potential variables from our questionnaire that we hypothesized might be associated with our outcome variable (Table 1). To help guard against obtaining unreliable estimates of parameters due to overfitting, we aimed to reduce our list of 18 variables so that no more than* m*/10 predictor terms would be included in each model, where* m *is the minimum of the number in either category of the outcome variable [22]. (Note that a variable with *k* categories would contribute  *k* − 1 predictor terms to the model.)

To avoid bias and inflation of type I error rates, reduction of the characteristics was carried out before we examined any bivariate (or any other) relationships between the potential exposure variables and the outcome variable. Reduction of the characteristics was based on our prior knowledge of the subject matter (prioritizing variables previously found to be associated with the outcome variable), observed distributions (variables with narrow distributions in which >90% of the responses were in one category or variables with >10% missing data were excluded), multicollinearity diagnostics (a variance inflation factor >2.5 was interpreted as evidence of collinearity between two exposure variables, so we chose the variable that had the stronger association with the outcome variable), and evaluation for confounding.

Based on this analysis plan, we reduced our list of 18 potential exposure variables to 8 variables (represented by 8 predictor terms, Table 2) for the analysis with men as there were 80 men whose most recent sexual partner was using hormonal or intrauterine contraception (Table 1). For our model for women, we reduced our list of 18 potential variables to 11 variables (represented by 11 predictor terms, Table 3) as there were 115 women using hormonal or intrauterine contraception at last intercourse.

## 3. Results and Discussion

We screened 349 women and 274 men and enrolled a total of 562 (308 women and 254 men) participants. We excluded 19 women from this analysis: 16 had undergone bilateral tubal ligation and 3 had missing contraceptive data (none had a partner who had undergone vasectomy). We also excluded 13 men from our analysis, all of whom had a partner who had undergone bilateral tubal ligation (no men enrolled in the study had undergone vasectomy). Therefore, our final analysis population included 289 women and 241 men.

The men and women in our study population significantly differed in seven out of the 17 variables that we examined (Table 1). The men had more children and were older, but they were less likely to report a history of an unplanned pregnancy and that the female made decisions about FP alone. However, the men were more likely to report that neither they nor their partner wanted to have more children, that they knew their most recent partner was HIV positive, that they had talked to their partner about which contraceptive to use, and that they had used a condom at last intercourse. Thirty-nine percent of women reported that they were using hormonal or intrauterine contraception at last intercourse, compared with 33.2% of men, which was not significantly different (*p* = 0.117). The men and women also did not significantly differ in education level, religion, current relationship status, residence, desire for another pregnancy in two years, length of time since HIV diagnosis, use of antiretroviral therapy, disclosure of HIV status to most recent partner, length of time with most recent partner, frequency of sex in the past month, use of other specific FP methods (excluding condoms), or use of hormonal or intrauterine contraception.

In the model for men, the only variable that was found to be predictive of hormonal or intrauterine contraceptive use by their most recent partner was the man having 3 or more children (Table 2, adjusted PR: 1.69; 95% CI: 1.11–2.59; *p* = 0.014). In the model for women, four variables were found to be predictive of hormonal or intrauterine contraceptive use at last intercourse (Table 3). Women using hormonal or intrauterine contraception were more likely to be younger (adjusted PR: 0.96; 95% CI: 0.94–0.98), less likely to want a pregnancy in two years (adjusted PR: 0.47; 95% CI: 0.31–0.72), more likely to have been with their most recent partner for four or more years (adjusted PR: 1.46; 95% CI: 1.02–2.09), and more likely to report that they alone made decision about FP (adjusted PR: 1.91, 95% CI: 1.32–2.77).

Some of the results for our analyses with women were similar to the findings from other studies, even though most of them include condom use as a contraceptive in their models. Younger age was also found to be associated with modern contraceptive use in two studies [7, 8]; these findings may suggest that younger women are more open to using modern contraceptives than previous generations. Having a regular partner (defined in our study as having sex more than four times a week) was also found to be associated with modern contraceptive use in three studies [9, 10, 17], which suggests that women who consider themselves to be of higher risk for pregnancy are more likely to use effective contraception. However, unlike other studies, we did not find higher education level, having three or more children, ART use, or disclosure of HIV status to partner to be associated with hormonal or intrauterine contraceptive use [7–12, 14–16]. The differences in findings may be due to the inclusion of condoms and permanent methods in some of the other studies or due to differences in our study populations.

It was encouraging to see that pregnancy intention for the next two years was associated with hormonal and intrauterine contraceptive use, suggesting that women who want to limit or delay future childbearing are getting the appropriate contraceptive counseling and access they need to meet their fertility goals. It was also reassuring to find that ART use was not associated with a decrease in hormonal and intrauterine contraceptive use, despite concerns about drug-drug interactions between certain ART with pills and implants [23]. However, this study was performed before several of the more recent studies related to ART-implant interactions were published, so it may not be reflective of current hormonal and intrauterine contraceptive use among women using ART in Malawi [24–27]. Ongoing evaluation of the impact of ART on contraceptive choice is warranted.

The differing results for variables associated with hormonal and intrauterine contraceptive use among the men and women in our study may be a result of the differences in their study population. As noted earlier, the men were significantly older than the women, with only one man below 25 years of age. Similarly, they were almost twice as likely to already have five or more children compared to women; ideal family size for both men and women in Malawi has been reported to be around 4 children [3].

Strengths of our study include our relatively large sample size, which allowed us to assess many variables in our models, and the fact that we surveyed both men and women and were able to compare the differences between them. However, the men and women in our survey were not necessarily couples and were not interviewed as couple dyads, so we could not corroborate the responses that the participants gave with their partners. This limitation is particularly important for the men who may not always be aware of what contraceptive methods their partner is using and may only have been reporting their perceptions rather than actual use. In addition, social desirability bias may have led both men and women to overreport modern contraceptive use or other variables such as decision-making in FP use. Using the variable “FP use at last intercourse” instead of current FP use also has its limitations as the participants may no longer be in a relationship and using that contraceptive. However, since we only included men and women who had been sexually active within the past six months, we felt that the participants' contraceptive behavior at last intercourse was more indicative of future contraceptive practices, particularly for those who were not currently in a relationship. Finally, given that our study was limited only to men and women who were attending HIV testing and treatment clinic in Lilongwe and who were mostly urban dwellers, the results may not be generalizable to other populations outside of Malawi or in more rural places in Malawi where access to hormonal and intrauterine contraception may be more limited. Even so, this analysis is the first to examine potential factors that are related to hormonal and intrauterine contraceptive use among men.

The findings from our study can help Malawi to meet its goals for Prong 2 of its HIV Prevention Strategy, which focuses on the reduction of unplanned or unintended pregnancies among HIV-infected women and HIV provider-initiated FP [5]. Integrating FP counseling and provision into HIV care is a challenge for many HIV providers given the high volume of HIV clients, limited number of providers, and extra time required to individually counsel all clients about their fertility desires and FP needs [19]. Understanding that HIV-infected men with 3 or more children may be more interested in using hormonal and intrauterine contraception in their relationship can encourage HIV providers to target these men and their partners for discussions about their desired family size and using effective contraceptive methods to prevent unwanted pregnancy. Similarly, providers should be encouraged to talk to HIV-infected women about their pregnancy intentions for the next 2 years, with the knowledge that those who do not want another pregnancy within 2 years may be particularly receptive to initiating hormonal or intrauterine contraception and should undergo targeted FP counseling.

Future studies could consider interviewing both members of a couple separately and then matching and comparing their responses to evaluate if they reported similar behaviors and perceptions within the couple and assess which questions were answered differently. They could also further explore the complex role that male partners play in FP decision-making, given the strong association found for our variable about FP decision-making in our model for women. Our study found that women who made FP decisions alone were more likely to be using hormonal or intrauterine contraception compared to women who involved the male partner or had a male partner who made all the FP decisions. Because of a large amount of missing data for our variable about talking to the partner about which FP method to use, we were not able to include it in our models. But the interplay between FP decision-making and the role of discussing hormonal and intrauterine contraceptive use needs to be better understood, particularly since two other studies found that it was associated with modern contraceptive use [9, 10].

## 4. Conclusions

Among men, use of hormonal and intrauterine contraception in their most recent relationship was associated with having 3 or more children, whereas, among women, younger age, not wanting a pregnancy in 2 years, being with their partner for more than 4 years, and being able to make family planning decisions by themselves were associated with hormonal and intrauterine contraceptive use. Knowledge of these associations may help providers to better understand the contraceptive needs and preferences of HIV-infected men and women and improve their FP uptake through targeted FP counseling.

## Figures and Tables

**Table 1:** Baseline characteristics of participants (by sex).

Characteristic	Women (*N* = 289)	Men (*N* = 241)	*p* value^*∗*^
	*n* (%)	*n* (%)	
Age^*∗∗*^			<0.001^*∗∗∗*^
18–24 years	36 (12.5)	1 (0.4)	
25–34 years	151 (52.1)	84 (34.9)	
≥35 years	102 (35.3)	155 (64.3)	

Education			0.806
Completed primary school or less	141 (48.8)	115 (47.7)	
Completed some secondary school or more	148 (51.2)	126 (52.3)	

Religion^*∗∗*^			0.509
Catholic	60 (20.8)	52 (21.7)	
Protestant	204 (70.6)	166 (69.2)	
Muslim	23 (8.0)	18 (7.5)	
No religion	1 (0.4)	4 (1.7)	

Current relationship status			0.910
Married/committed relationship with 1 partner	281 (97.2)	236 (98.0)	
Dating one or more persons	5 (1.7)	3 (1.2)	
Not currently dating	3 (1.0)	2 (0.8)	

Residence^*∗∗*^			0.154
Urban	260 (90.0)	208 (86.3)	
Rural	28 (9.7)	33 (13.7)	

Number of children			0.014^*∗∗∗*^
0	23 (7.0)	9 (3.7)	
1-2	138 (47.8)	98 (40.7)	
3-4	100 (34.6)	95 (39.4)	
5 or more	28 (9.7)	39 (16.2)	

History of unplanned pregnancy^*∗∗*^			0.032^*∗∗∗*^
No	146 (50.5)	143 (59.3)	
Yes	143 (49.5)	96 (39.8)	

Wants pregnancy within the next 2 years^*∗∗*^			0.159
No	202 (69.9)	178 (73.9)	
Yes	87 (30.1)	58 (24.1)	

Neither you nor partner wants more kids^*∗∗*^			0.010^*∗∗∗*^
No	142 (49.1)	92 (38.2)	
Yes	144 (49.8)	147 (61.0)	

Length of time since HIV diagnosis^*∗∗*^			0.223
≤5 years	135 (46.7)	101 (41.9)	
>5 years	151 (52.2)	140 (58.1)	

On antiretroviral therapy			0.516
No	33 (11.4)	32 (13.3)	
Yes	256 (88.6)	209 (86.7)	

HIV status of most recent partner^*∗∗*^			0.044^*∗∗∗*^
Negative	56 (19.4)	44 (18.3)	
Positive	184 (63.7)	173 (71.8)	
Do not know	48 (16.6)	23 (9.5)	

Most recent partner knows your HIV status^*∗∗*^			0.790
No	18 (6.2)	12 (5.0)	
Yes	270 (93.4)	227 (94.2)	
Do not know	1 (0.4)	1 (0.4)	

Length of time with most recent partner^*∗∗*^			0.994
≤4 years	92 (31.8)	78 (32.4)	
>4 years	189 (65.4)	160 (66.4)	

In the past month, frequency of sex^*∗∗*^			0.370
≤4 times (once a week or less)	56 (19.4)	55 (22.8)	
>4 times (more than once a week)	129 (44.6)	103 (42.7)	

Talk to most recent partner about which FP method to use^*∗∗*^			0.008^*∗∗∗*^
Yes	19 (6.6)	191 (79.3)	
No	203 (70.2)	5 (2.1)	

Who makes decisions about family planning^*∗*^			<0.001^*∗∗∗*^
Female only	189 (65.4)	84 (34.9)	
Male only	38 (13.1)	66 (27.4)	
Both	40 (13.8)	62 (25.7)	

Have you ever learned about family planning from a healthcare provider?^*∗∗*^			N/A
No	17 (5.9)	N/A	
Yes	271 (94.8)	N/A	

Contraceptive(s) used at last intercourse			
No method	77 (26.6)	52 (21.6)	0.176
Natural family planning	1 (0.3)	0 (0)	N/A
Withdrawal	1 (0.3)	1 (0.4)	1.000
Condoms	167 (57.8)	161 (66.8)	0.033^*∗∗∗*^
Condoms only	96 (33.2)	108 (44.8)	0.006^*∗∗∗*^
Condoms plus another method	71 (24.6)	53 (22.0)	0.001^*∗∗∗*^
Oral contraceptive	14 (4.8)	10 (4.1)	0.689
Injection	67 (23.1)	43 (17.8)	0.121
Implant	31 (10.7)	25 (10.4)	0.874
Intrauterine device	3 (1.0)	2 (0.8)	0.582
Emergency contraception	0 (0)	0 (0)	N/A
Hormonal or intrauterine contraception	115 (39.8)	80 (33.2)	0.117

^*∗*^ *p* value calculated using Pearson's chi-squared test or Fisher's exact test.

^*∗∗*^Contains missing data so percentages may not add up to 100%.

^*∗∗∗*^Statistically significant with *p* < 0.05.

**Table 2:** Unadjusted and adjusted prevalence ratios for hormonal or intrauterine contraceptive use at last intercourse among 241 HIV-infected men.

Variable (*N*)	*n* (%) using modern FP	Unadjusted PR^a^ (95% CI)	Adjusted PR^b^ (95% CI)	*p* value^c^
Age (*N* = 240, continuous)	N/A	1.00 (0.97–1.04)	0.97 (0.94–1.01)	0.117

Education (*N* = 241)				0.366
Completed primary school or less	43 (37.4)	—	—	
Completed some secondary school or more	37 (29.4)	0.79 (0.55–1.13)	0.84 (0.58–1.22)	

Religion (*N* = 241)				0.919
Not Catholic (Protestant/Muslim/none)	63 (33.3)	—	—	
Catholic	14 (32.7)	0.98 (0.63–1.52)	1.02 (0.67–1.55)	

Number of children (*N* = 241)				0.014^*∗*^
0–2	26 (24.3)	—	—	
3 or more	54 (40.3)	1.65 (1.12–2.46)	1.69 (1.11–2.59)	

History of unplanned pregnancy (*N* = 239)				0.121
No	50 (35.0)	—	—	
Yes	29 (30.2)	0.86 (0.59–1.26)	0.74 (0.50–1.08)	

Wants pregnancy within the next 2 years (*N* = 236)				0.081
No	67 (37.6)	—	—	
Yes	13 (22.4)	0.60 (0.36–1.00)	0.61 (0.35–1.06)	

HIV status of most recent partner (*N* = 240)				0.914
Negative	14 (31.8)	—	—	
Positive/do not know	66 (33.7)	1.06 (0.66–1.70)	1.03 (0.64–1.66)	

Length of time with most recent partner (*N* = 238)				0.108
≤4 years	19 (24.4)	—	—	
>4 years	61 (38.1)	1.57 (1.01–2.43)	1.44 (0.92–2.23)	

^a^Unadjusted results from modified Poisson regression models.

^b^Adjusted results from modified Poisson regression model (*N* = 232 observations with nonmissing values on all independent variables), adjusting for all other variables in Table 2.

^c^ *p* value from Poisson regression model adjusting for all variables in Table 2.

^*∗*^Statistically significant with *p* < 0.05.

**Table 3:** Unadjusted and adjusted prevalence ratios for hormonal or intrauterine contraceptive use at last intercourse among 289 HIV-infected women.

Variable (*N*)	% using modern family planning	Unadjusted PR^a^ (95% CI)	Adjusted PR^b^ (95% CI)	*p* value^c^
Age (*N* = 289, continuous)	N/A	0.98 (0.96–1.01)	0.96 (0.94–0.98)	0.001^*∗*^

Education (*N* = 289)				0.301
Completed primary school or less	50 (35.5)	—	—	
Completed some secondary school or more	65 (43.9)	1.24 (0.93–1.65)	1.16 (0.87–1.55)	

Religion (*N* = 289)				0.385
Not Catholic (Protestant/Muslim/none)	89 (38.9)	—	—	
Catholic	26 (43.3)	1.11 (0.80–1.55)	1.10 (0.81–1.48)	

Number of children (*N* = 289)				0.385
0–2	61 (37.9)	—	—	
3 or more	54 (42.2)	1.11 (0.84–1.48)	1.15 (0.84–1.55)	

History of unplanned pregnancy (*N* = 289)				0.643
No	61 (41.8)	—	—	
Yes	54 (37.8)	0.90 (0.68–1.20)	0.94 (0.71–1.24)	

Wants pregnancy in next 2 years (*N* = 289)				<0.001^*∗*^
No	96 (47.5)	—	—	
Yes	19 (21.8)	0.46 (0.30–0.70)	0.47 (0.31–0.72)	

Length of time since HIV diagnosis (*N* = 286)				0.528
≤5 years	54 (40.0)	—	—	
>5 years	60 (39.7)	0.99 (0.75–1.32)	0.91 (0.69–1.21)	

On antiretroviral therapy (*N* = 289)				0.498
No	13 (39.4)	—	—	
Yes	102 (39.8)	1.01 (0.64–1.59)	0.87 (0.58–1.30)	

HIV status of most recent partner (*N* = 289)				0.595
Negative	22 (39.3)	—	—	
Positive/do not know	93 (40.1)	1.02 (0.71–1.47)	0.92 (0.66–1.26)	

Length of time with most recent partner (*N* = 281)				0.037^*∗*^
≤4 years	28 (30.4)	—	—	
>4 years	85 (45.0)	1.48 (1.04–2.09)	1.46 (1.02–2.09)	

Who makes decisions about family planning (*N* = 289)				0.001^*∗*^
Female only	89 (47.1)	1.81 (1.26–2.61)	1.91 (1.32–2.77)	
Male only/both female and male	26 (26.0)	—	—	

^a^Unadjusted results from modified Poisson regression models.

^b^Adjusted results from modified Poisson regression model (*N* = 277 observations with nonmissing values on all independent variables), adjusting for all other variables in Table 3.

^c^ *p* value from Poisson regression model adjusting for all variables in Table 3.

^*∗*^Statistically significant with *p* < 0.05.

## References

[1] World Health Organization (2015). *WHO HIV/AIDS Fact Sheet, #360*.

[2] UNAIDS (2014). *UNAIDS Malawi HIV/AIDS Estimates (2014)*.

[3] National Statistical Office (NSO) (2011). *Malawi Demographic and Health Survey 2010*.

[4] Ministry of Health (2011). *Clinical Management of HIV in Children and Adults*.

[5] National AIDS Commission (2014). *National HIV Prevention Strategy, 2015–2020*.

[6] Haddad L., Phiri S., Cwiak C. (2011). Fertility preferences, unintended pregnancy and contraceptive use among HIV-positive women desiring family planning in Lilongwe, Malawi. *Contraception*.

[7] Muyindike W., Fatch R., Steinfield R. (2012). Contraceptive use and associated factors among women enrolling into HIV care in southwestern Uganda. *Infectious Diseases in Obstetrics and Gynecology*.

[8] Chibwesha C. J., Li M. S., Matoba C. K. (2011). Modern contraceptive and dual method use among HIV-infected women in Lusaka, Zambia. *Infectious Diseases in Obstetrics and Gynecology*.

[9] Melaku Y. A., Zeleke E. G. (2014). Contraceptive utilization and associated factors among HIV positive women on chronic follow up care in tigray region, northern ethiopia: a cross sectional study. *PLoS ONE*.

[10] Polis C. B., Gray R. H., Lutalo T. (2011). Trends and correlates of hormonal contraceptive use among HIV-infected women in Rakai, Uganda, 1994–2006. *Contraception*.

[11] Polisi A., Gebrehanna E., Tesfaye G., Asefa F. (2014). Modern contraceptive utilization among female ART attendees in health facilities of Gimbie town, West Ethiopia. *Reproductive Health*.

[12] Asfaw H. M., Gashe F. E. (2014). Contraceptive use and method preference among HIV positive women in Addis Ababa, Ethiopia: a cross sectional survey. *BMC Public Health*.

[13] Nieves C. I., Kaida A., Seage G. R. (2015). The influence of partnership on contraceptive use among HIV-infected women accessing antiretroviral therapy in rural Uganda. *Contraception*.

[14] Andia I., Kaida A., Maier M. (2009). Highly active antiretroviral therapy and increased use of contraceptives among HIV-positive women during expanding access to antiretroviral therapy in Mbarara, Uganda. *American Journal of Public Health*.

[15] Kaida A., Laher F., Strathdee S. A. (2010). Contraceptive use and method preference among women in Soweto, South Africa: the influence of expanding access to HIV care and treatment services. *PLoS ONE*.

[16] Laryea D. O., Amoako Y. A., Spangenberg K., Frimpong E., Kyei-Ansong J. (2014). Contraceptive use and unmet need for family planning among HIV positive women on antiretroviral therapy in Kumasi, Ghana. *BMC Women's Health*.

[17] Ezugwu E. C., Nkwo P. O., Agu P. U., Ugwu E. O., Asogwa A. O. (2014). Contraceptive use among HIV-positive women in Enugu, southeast Nigeria. *International Journal of Gynecology & Obstetrics*.

[18] World Health Organization (2011). *Knowledge for Health Project. Family Planning: A Global Handbook for Providers (2011 update)*.

[19] Phiri S., Feldacker C., Chaweza T. (2016). Integrating reproductive health services into HIV care: strategies for successful implementation in a lowresource HIV clinic in Lilongwe, Malawi. *Journal of Family Planning and Reproductive Health Care*.

[20] Zou G. Y. (2004). A modified poisson regression approach to prospective studies with binary data. *American Journal of Epidemiology*.

[21] Coutinho L. M. S., Scazufca M., Menezes P. R. (2008). Methods for estimating prevalence ratios in cross-sectional studies. *Revista de Saúde Pública*.

[22] Harrell F. E. (2001). *Regression Modeling Strategies: With Applications to Linear Models, Logistic Regression, and Survival Analysis*.

[23] World Health Organization (2015). *Medical Eligibility Criteria*.

[24] Perry S. H., Swamy P., Preidis G. A., Mwanyumba A., Motsa N., Sarero H. N. (2014). Implementing the Jadelle implant for women living with HIV in a resource-limited setting: concerns for drug interactions leading to unintended pregnancies. *AIDS*.

[25] Pyra M., Heffron R., Mugo N. R. (2015). Effectiveness of hormonal contraception in HIV-infected women using antiretroviral therapy. *AIDS*.

[26] Patel R. C., Onono M., Gandhi M. (2015). Pregnancy rates in HIV-positive women using contraceptives and efavirenz-based or nevirapine-based antiretroviral therapy in Kenya: a retrospective cohort study. *The Lancet HIV*.

[27] Scarsi K. K., Darin K. M., Nakalema S. (2016). Unintended pregnancies observed with combined use of the levonorgestrel contraceptive implant and efavirenz-based antiretroviral therapy: a three-arm pharmacokinetic evaluation over 48 weeks. *Clinical Infectious Diseases*.

